# Lassa antiviral LHF-535 protects guinea pigs from lethal challenge

**DOI:** 10.1038/s41598-022-23760-2

**Published:** 2022-11-19

**Authors:** Kathleen A. Cashman, Eric R. Wilkinson, Jeffrey Posakony, Ikenna G. Madu, Eric J. Tarcha, Kurt H. Lustig, Marcus J. Korth, Kristin M. Bedard, Sean M. Amberg

**Affiliations:** 1 Virology Division, U.S. Army Medical Research Institute of Infectious Diseases (USAMRIID), Fort Detrick, Frederick, MD USA; 2 Kineta, Inc., Seattle, WA USA; 3 Present Address: AltPep, Seattle, WA USA; 4 Present Address: Lyell Immunopharma, Seattle, WA USA; 5 Present Address: Sutro Biopharma, South San Francisco, CA USA

**Keywords:** Drug discovery, Microbiology

## Abstract

LHF-535 is a small molecule antiviral currently in development for the treatment of Lassa fever, a zoonotic disease endemic in West Africa that generates significant morbidity and mortality. Current treatment options are inadequate, and there are no approved therapeutics or vaccines for Lassa fever. LHF-535 was evaluated in a lethal guinea pig model of Lassa pathogenesis, using once-daily administration of a fixed dose (50 mg/kg/day) initiating either 1 or 3 days after inoculation with a lethal dose of Lassa virus. LHF-535 reduced viremia and clinical signs and protected all animals from lethality. A subset of surviving animals was rechallenged four months later with a second lethal challenge of Lassa virus and were found to be protected from disease. LHF-535 pharmacokinetics at the protective dose in guinea pigs showed plasma concentrations well within the range observed in clinical trials in healthy volunteers, supporting the continued development of LHF-535 as a Lassa therapeutic.

## Introduction

Lassa fever, an acute viral hemorrhagic fever disease endemic in West Africa, is responsible for a significant disease burden. While the true number of cases is uncertain, public health officials often cite an estimated impact of several hundred thousand cases and several thousand deaths annually^1,2^. One recent study suggests that Lassa virus, a member of the family *Arenaviridae* and the etiologic agent of Lassa fever, is one of the highest known zoonotic spillover threats^3^. The current approach to treating Lassa fever is supportive therapy that is often combined with off-label use of the broad-spectrum antiviral drug ribavirin. However, the efficacy of ribavirin for treating Lassa fever is unproven, and outbreaks of the disease are often associated with high rates of mortality, even among ribavirin-treated patients^4^. This was acutely evident during a recent outbreak in Nigeria in which the case fatality rate was 21% among patients hospitalized with a confirmed case of Lassa fever and treated with ribavirin^5^. Recent analyses have questioned the data supporting the clinical effectiveness of ribavirin and have suggested that ribavirin may actually be detrimental, particularly in milder cases^6,7^. To address the need for better therapeutic options, we are developing LHF-535, a small-molecule antiviral drug candidate that targets the envelope glycoprotein of Lassa virus.

LHF-535 is an analog of the previously characterized benzimidazole derivative ST-193^8–11^, which acts as an antiviral drug by inhibiting arenavirus entry into host cells. Entry is a multi-step process that is mediated by the viral envelope glycoprotein complex, which consists of a receptor-binding subunit (GP1), a transmembrane fusion subunit (GP2), and a stable signal peptide (SSP) that interacts with GP2^12^. After GP1 binds to a cell surface receptor, the virus is endocytosed and GP2 undergoes a pH-dependent conformational rearrangement that facilitates fusion of the viral and endosomal membranes. LHF-535 and ST-193 are thought to bind to and stabilize an SSP-GP2 prefusion structure, thereby suppressing the rearrangement of GP2 that is necessary for membrane fusion.

LHF-535 has been optimized for pharmacokinetic properties, antiviral potency, and broad-spectrum activity against arenaviruses. The compound has potent activity against lentiviral pseudotype viruses expressing envelope glycoproteins from across the Lassa virus phylogeny or from New World clade B arenaviruses associated with hemorrhagic fever, such as Junín, and Machupo^13^. In addition, a daily oral dose of LHF-535 at 10 mg/kg protects AG129 mice from a lethal dose of Tacaribe virus, an arenavirus closely related to Junín virus^13^.

Although chimeric, related, or attenuated viruses are useful for early evaluation of antiviral therapies or for investigating aspects of arenavirus pathogenesis, studies using viruses that are authentic human pathogens provide important validation. Here, we evaluated the antiviral efficacy of LHF-535 in a well-characterized guinea pig model of Lassa fever. In this model, infection of strain 13 guinea pigs with Lassa virus results in a uniformly fatal disease that is characterized by fever, weight loss, interstitial pneumonia, and high viral titers in the lung, spleen, and lymph nodes^14,15^. This small animal model has been used extensively to characterize candidate medical countermeasures^8,16,17^. We show that LHF-535 protected 100% of guinea pigs infected with a lethal dose of Lassa virus, even when treatment was initiated 3 days after infection. In addition to surviving the initial infection, LHF-535-treated animals developed protective immunity against rechallenge. These findings support further development of LHF-535 as a new treatment for Lassa fever.

## Results

### LHF-535 protects guinea pigs against lethal Lassa virus infection

Strain 13 guinea pigs were used to evaluate the antiviral efficacy of LHF-535 when administered 24 or 72 h after infection with a lethal dose of Lassa virus. All animals were acclimated for 3 days in BSL-4 animal housing prior to the start of the study and on Day 0 were infected with a target dose of 1000 pfu of guinea pig-adapted Lassa virus (Josiah strain) by subcutaneous injection. One animal in the control group (Group 1) died after reviving from anesthesia and was excluded from analysis. The remaining animals in the control group received a daily dose of vehicle alone beginning on Day 1 after infection. Animals in the experimental groups received a daily dose of LHF-535 (50 mg/kg) beginning on Day 1 (Group 2) or Day 3 (Group 3) after infection. All surviving animals in each group continued to receive daily treatment until Day 22.

**Figure 1:**
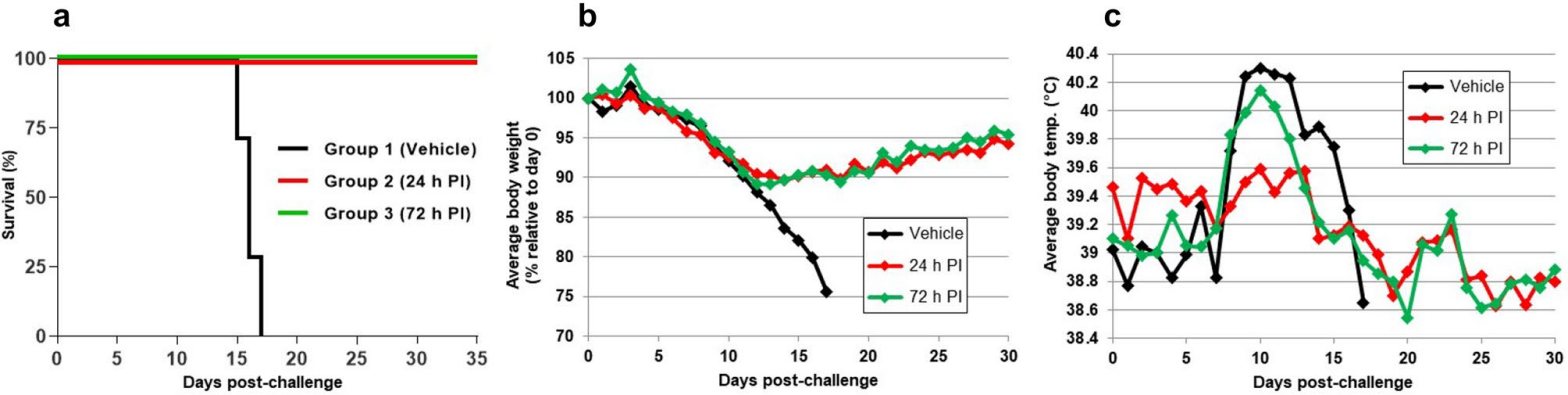
Study outcomes. (**a**) Survival by group (daily LHF-535 administration initiated 24 h post-infection (PI) for group 2 and 72 h PI for group 3); (**b**) Average body weight; and (**c**) Average body temperature. Body temperature recorded in one group 3 animal was consistently 2.5 °C lower than the other animals in the group and was excluded from the averages shown here; had it been included, average group 3 temperatures would be 0.3 °C lower.

**Figure 2:**
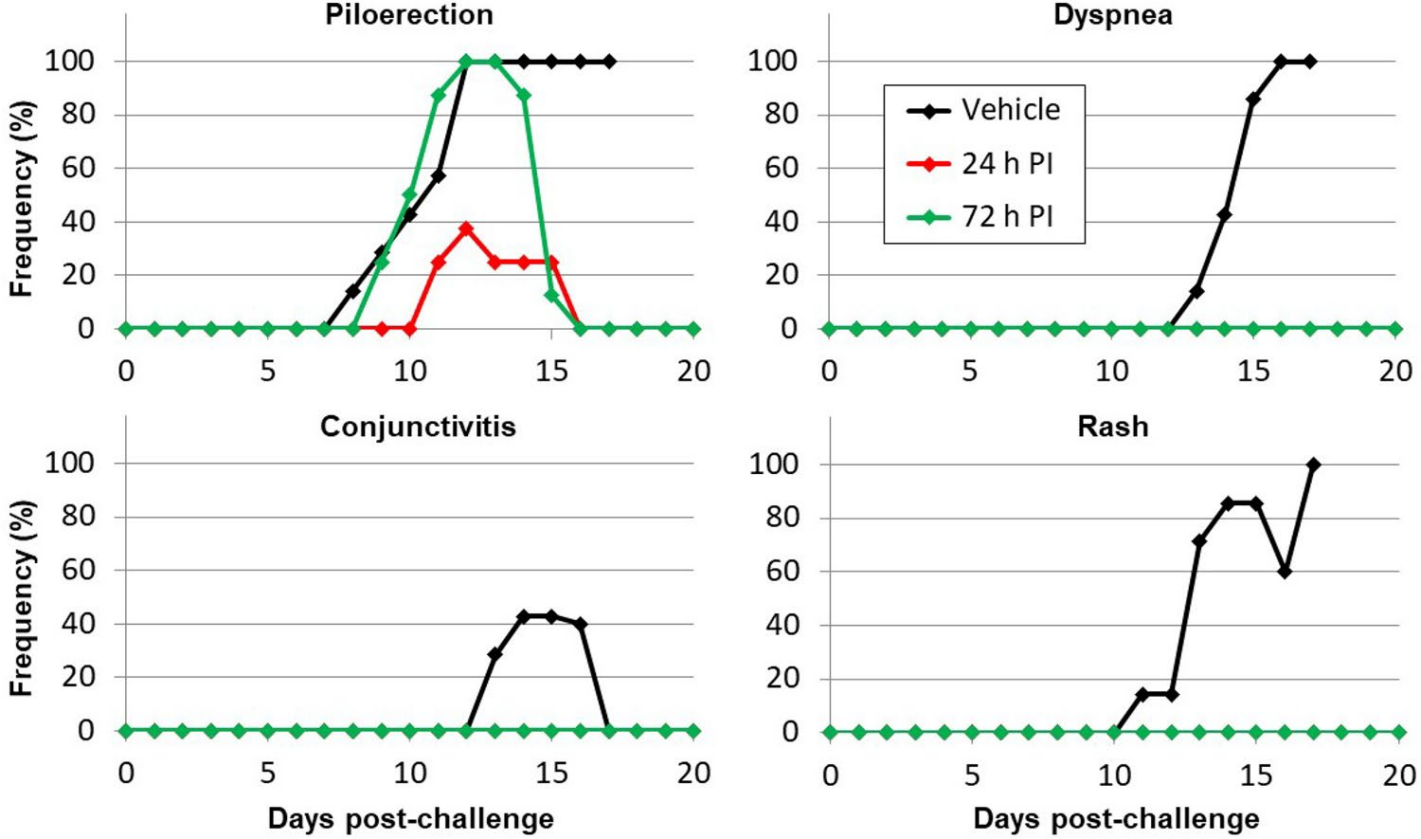
Frequency of clinical signs observed following viral challenge by group.

Animals in the control group exhibited progressive weight loss (> 20%), became febrile between Day 8 and Day 10 (body temperature > 39.5 °C), and succumbed to Lassa virus infection between Day 15 and Day 17 (Fig. 1), with a mean time-to-death (MTD) of 16.0 days. Animals treated with LHF-535 initially lost weight and became febrile, but weight loss stabilized by Day 12, and for most animals, fevers resolved by Day 14. All animals treated with LHF-535 survived the infection. Clinical observations for animals in the control group were more frequent than for animals treated with LHF-535 (Fig. 2). The majority of animals in the control group became lethargic, developed a rough coat and rash, and exhibited labored breathing. These clinical signs were absent from animals treated with LHF-535, although transient piloerection was noted.

### LHF-535 reduces serum viremia

**Figure 3:**
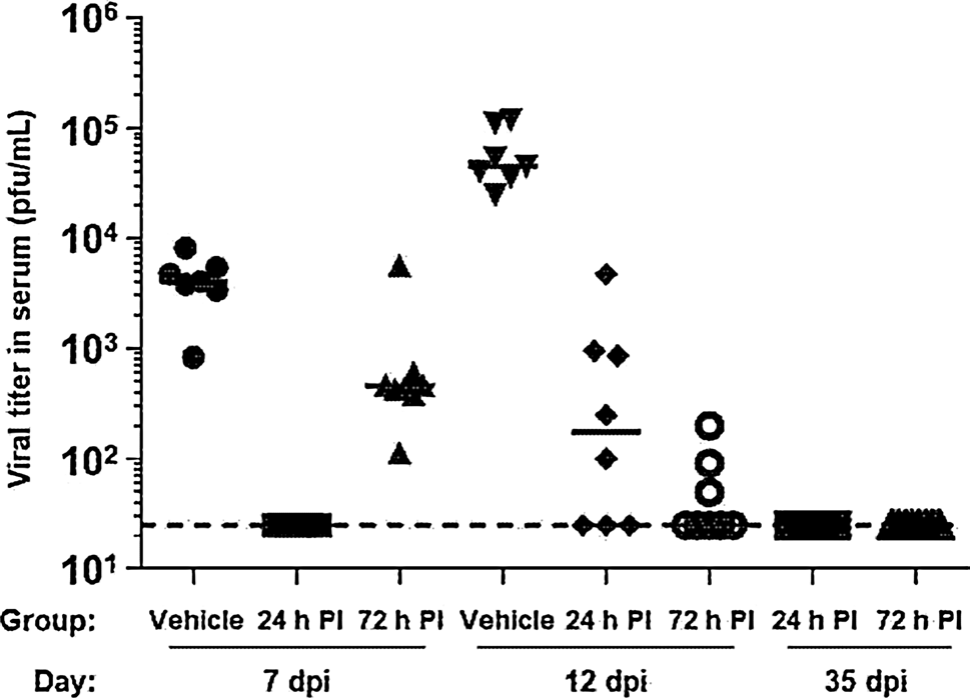
Viral titers in serum at 7, 12, and 35 days post-infection. The limit of detection (25 pfu/mL) is indicated by the dashed line and short horizontal bars show group medians.

All animals in Group 1 and Group 3 became viremic after infection (Fig. 3). At Day 7, the average (geometric mean) viremia was 2.8 × 10^3^ pfu/ml in the control group and 4.1 × 10^2^ pfu/ml in Group 3. This difference was statistically significant (*p* < 0.001). In contrast, no virus was detected in the serum of animals that received LHF-535 1 day after infection (Group 2). At Day 12, the average viremia was 4.8 × 10^4^ pfu/ml in the control group, whereas virus was detected in the serum of only 5 of 8 animals in Group 2, and 3 of 8 animals in Group 3. The differences between groups 1 and 3 and the control group were statistically significant (*p* < 0.001). No virus was detected in the serum of any LHF-535-treated animal at the study endpoint (Day 35 after infection).

### Surviving LHF-535-treated animals are immune to Lassa virus rechallenge

Previous studies have demonstrated that guinea pigs that survive Lassa virus infection develop neutralizing antibodies to the virus^8,15^. To test whether surviving LHF-535-treated animals develop protective immunity against rechallenge, eight of the surviving animals were held until 120 days after infection (7.5 times the MTD of the vehicle-treated animals), at which time they were again challenged with a lethal dose of Lassa virus. As a control group, eight age-matched naive animals were infected with Lassa virus in the same manner.

**Figure 4:**
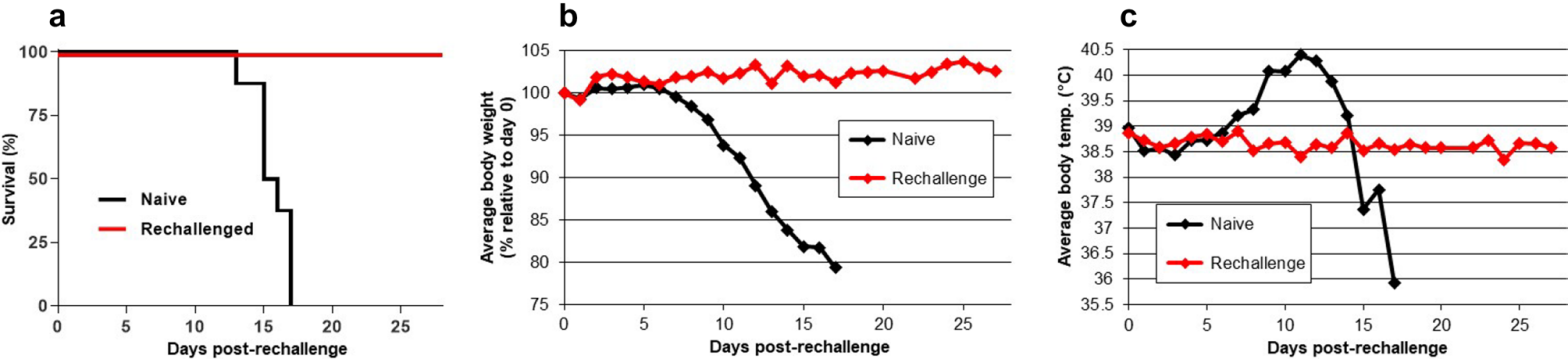
Study outcomes following rechallenge. (**a**) Survival by group, comparing outcome of rechallenged animals with age-matched naive controls; (**b**) Average body weight; and (**c**) Average body temperature. Body temperature recorded in one rechallenged animal was consistently 2.5 °C lower than the other animals in the group and was excluded from the averages shown here; had it been included, average rechallenged animal temperatures would be 0.3 °C lower.

All eight of the LHF-535-treated animals that survived the initial infection were protected against rechallenge and exhibited no significant change in body weight or temperature over a 28-day period after infection (Fig. 4). In contrast, animals in the naive control group exhibited progressive weight loss and became febrile, and there was 100% mortality in this group by 17 days after infection, with an MTD of 15.6 days. There were no clinical observations in the rechallenged group except for a single note of piloerection in one animal at ten days post-rechallenge. LHF-535 therefore protected guinea pigs from a lethal dose of Lassa virus, and all surviving animals developed protective immunity against rechallenge with the same virus.

### Pharmacokinetics of LHF-535 in guinea pigs

**Figure 5:**
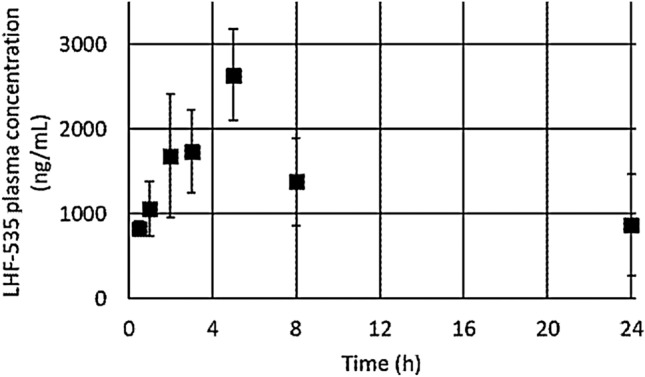
LHF-535 pharmacokinetics following intraperitoneal administration at a dose of 50 mg/kg in Hartley guinea pigs. Markers indicate average plasma concentration of three animals and error bars denote standard deviation.

The pharmacokinetics of LHF-535 was evaluated from a single intraperitoneal dose of 50 mg/kg in healthy outbred Hartley guinea pigs using the same formulation as described. Plasma LHF-535 reached a C_max_ of 2637 ng/mL at a T_max_ of 5 h, generating an average 24-h area under the plasma concentration curve (AUC_0–24 h_) of 31.7 μg·h/mL (Fig. 5). Intraperitoneal administration of other LHF-535 formulations in guinea pigs have exhibited similar pharmacokinetics. Repeat daily intraperitoneal dosing showed similar AUC_0–24 h_ values after 13 or 20 days (median 50.7 μg·h/mL, N = 8) compared to AUC_0–24 h_ values after the first dose (median 51.0 μg·h/mL, N = 12). Similarly, plasma concentrations at 24 h post-dosing (C_24_, equivalent to C_min_ or trough exposure) following the first dose (average 485 ng/mL, median 243 ng/mL, N = 20) are comparable to those after 7 to 20 daily doses (average 426 ng/mL, median 253 ng/mL, N = 16).

## Discussion

There are no approved drugs or vaccines to treat or protect against Lassa fever, and the World Health Organization has identified the disease as a top priority for research and development efforts^18^. Here, we showed that the antiviral drug candidate LHF-535, a small-molecule inhibitor of arenavirus entry, protected strain 13 guinea pigs from a lethal dose of Lassa virus. LHF-535 is an optimized analog of ST-193, which was previously evaluated for antiviral activity against Lassa virus in the strain 13 guinea pig model^8^. In the prior study, ST-193 was administered by intraperitoneal injection at a dose of 25 or 80 mg/kg/day beginning 1 h prior to a lethal dose of Lassa virus. Both doses of ST-193 provided a similar level of protection, and the overall survival rate for animals treated with ST-193 was 62.5%. The 100% survival rate for LHF-535-treated animals may be due to the increased potency of the optimized drug candidate^13^.

In addition to surviving Lassa virus infection, LHF-535-treated animals showed fewer clinical signs of disease, including reduced fever, weight loss, and viremia relative to animals treated with vehicle alone. The reduction in viremia was particularly evident, and 3 of 8 animals that received LHF-535 1 day after infection had no detectable virus at any time point evaluated. No infectious virus was detected in the serum of any LHF-535-treated animal at the endpoint of the study. However, more sensitive methods such as RT-PCR might have shown the presence of viral RNA; also, we cannot rule out the possibility of viral persistence in a non-blood compartment such as the central nervous system. The ability of LHF-535 to reduce viremia may be important for treating patients with Lassa fever, where high viral load directly correlates with poor clinical outcome^19–21^. The effect of LHF-535 on viral load is also in contrast to that of ribavirin, which often mediates only modest effects on viremia in animal models of Lassa fever^8,22^. In immunocompromised mouse models of Lassa virus infection, ribavirin appears to act by protecting infected cells from dying, which may result in reduced damage to liver tissue^22,23^.

ST-193-treated guinea pigs that survive Lassa virus infection develop a Lassa-virus-specific IgG2 response starting 21 days after infection, and serum samples from these animals have neutralizing activity against the virus^8^. Similarly, out-bred Hartley guinea pigs that survive Lassa virus infection also develop neutralizing antibody activity late in convalescence (> 32 days after infection)^15^. However, the question of whether surviving animals develop protective immunity against rechallenge has not been examined. Here, we showed that surviving LHF-535-treated animals were immune to Lassa virus rechallenge and exhibited no significant change in body weight or temperature after infection.

In humans, survival from Lassa virus infection is thought to produce life-long protective immunity^24^. Seropositive individuals have Lassa-virus-specific CD4^+^ T cells, and a T cell response is considered to be essential for controlling Lassa virus infection^25,26^. Although a neutralizing humoral response is also present in convalescent serum from patients surviving Lassa fever^27^, the role of neutralizing antibodies in controlling human infections is less clear. High antibody titer does not correlate with recovery^21^, and passive transfer of survivor plasma does protect against Lassa virus infection^28^. However, a cocktail of human monoclonal antibodies that target the Lassa virus glycoprotein provides protection in guinea pig and nonhuman primate models of Lassa fever^29,30^.

LHF-535 protected 100% of guinea pigs when treatment was initiated 3 days after infection. The full duration of the effective treatment window is not known, and alternative dosing regimens remain to be examined. The ability to initiate successful treatment multiple days after infection will be important for treating Lassa fever in humans, where the time from infection to initiation of care is highly variable^19^. Treatment with ribavirin, the current standard of care for Lassa fever, has been called into serious question^6,7^. The pharmacokinetics of LHF-535 in guinea pigs corresponding to an efficacious dosing regimen in a lethal challenge model will be informative for guiding a target clinical exposure. Phase I clinical trials of LHF-535 in healthy volunteers, using both single and 14-day dosing, established that exposures in excess of the guinea pig exposures reported here can be safely achieved (manuscript in preparation). Taken together, these results support the further development of LHF-535 as a new treatment for Lassa fever.

## Methods

### Ethics statement

Research was conducted at the United States Army Medical Research Institute of Infectious Diseases (USAMRIID). USAMRIID’s Institutional Animal Care and Use Committee (IACUC) approved the protocol in compliance with the Animal Welfare Act, Public Health Service (PHS) assurance, and all other Federal statutes and regulations relating to animals and experiments involving animals. The USAMRIID facility is accredited by the Association for Assessment and Accreditation of Laboratory Animal Care International (AAALAC) and adheres to principles stated in the Guide for the Care and Use of Laboratory Animals, National Research Council, 2011.

### Biosafety

All work with Lassa virus and potentially infectious materials derived from animals was conducted in a biosafety level 4 (BSL-4) laboratory. Virus inactivation prior to the removal of samples from the BSL-4 laboratory was performed according to USAMRIID standard operating procedures.

### Animals

Male and female strain 13 guinea pigs (*Cavia porcellus*) were obtained from the USAMRIID in-house colony; animals ranged in age from 3 to 5 months and in weight from 570 to 780 g at study initiation. Five days prior to the start of the study, the animals were implanted subcutaneously with microchip transponders for identification and temperature measurement. Animals were randomized into 3 groups of 8 animals each, and groups were balanced by weight and sex to minimize bias. Animals were offered standard guinea pig feed and water ad libitum*,* as well as daily enrichments such as spinach, alfalfa, or fruit. Animal procedures were performed in accordance with recommended set of ARRIVE guidelines.

### Virus, challenge inoculum, and plaque assay

A guinea-pig-adapted clinical isolate of Lassa virus (Josiah strain) was used for these studies. The virus originated from a fatal human case of Lassa fever and was adapted to provide uniform lethality in strain 13 guinea pigs^14,15^. The challenge stock was prepared by propagation in Vero cell culture and screened by polymerase chain reaction using primer sets specific for a variety of to potential contaminating viruses, transmission electron microscopy to evaluate virus particle integrity, endotoxin, mycoplasma, and bacterial contamination assessments, and deep sequencing to identify viral quasi species. The challenge inoculum was prepared by diluting the challenge stock in sterile 0.9% normal saline. The titers of the challenge stock, dilution series, and challenge inoculum were determined by using a neutral-red-based Vero cell plaque assay^14^. Viral titers in serum collected from animals during the course of the study were also determined by this method.

### LHF-535

LHF-535 is a small-molecule compound of the bis-substituted benzimidazole class^13^. A stock suspension of micronized LHF-535 was prepared at 10 mg/ml in a vehicle consisting of 0.5% Methocel E15 LV (DuPont) and 1% Tween 80 and stored at 4 °C until use. Micronization is used to reduce particle size, improve dissolution rate, and enhance reproducibility. Before use, the suspension was briefly sonicated and allowed to equilibrate to room temperature. Control formulation (vehicle alone) was prepared and stored in parallel.

### Antiviral efficacy and rechallenge studies

Guinea pigs were randomized into 3 groups of 8 animals each (4 males and 4 females) and moved to BSL-4 containment 3 days prior to the start of the study (Day − 3). On Day 0, animals were anesthetized and subcutaneously injected with a target dose of 1,000 plaque forming units (pfu) of Lassa virus in 200 μl of normal saline. Animals in the vehicle control group (Group 1) received a daily intraperitoneal injection of formulation buffer alone (5 ml/kg) beginning on Day 1. Animals in the remaining groups received a daily intraperitoneal injection of LHF-535 (50 mg/kg) beginning on Day 1 (Group 2) or Day 3 (Group 3) after infection. For all groups, surviving animals continued to receive daily treatment through Day 22. Animals were observed twice daily and clinical signs were recorded, including piloerection; anorexia, dehydration, or visible weight loss (≥ 10%); rash; orbital exudates (conjunctivitis); ataxia; and dyspnea (labored breathing). Body weight and temperature measurements were recorded daily. Blood samples were collected at Days 0, 7, 12, and at euthanasia. Animals were anesthetized by intramuscular injection of a KAX cocktail (60.6 mg/mL ketamine HCl, 0.6 mg/mL acepromazine maleate, and 6.67 mg/mL xylazine HCl) prior to sampling (0.2 mL KAX) or euthanasia procedures (0.3 mL KAX). Animals were euthanized when humane endpoint criteria (non-ambulatory, respiratory distress, hypothermia, excessive body weight loss) were met in accordance with the IACUC-approved protocol, or at the scheduled study endpoint.

The efficacy study was ended at Day 35. Eight of the surviving LHF-535-treated animals were held until 120 days after infection (4 from each of the two LHF-535 treatment groups), at which time they were rechallenged with a lethal dose of Lassa virus. Eight age-matched naive animals were infected with Lassa virus in the same manner. The methods for virus infection and monitoring of animals after infection were the same as described for the efficacy study. The rechallenge study was ended 28 days after rechallenge with Lassa virus.

### Pharmacokinetics

LHF-535 was prepared as described (10 mg/ml) and administered to female Hartley guinea pigs (N = 6) by intraperitoneal injection using a dosing volume of 5 ml per kg body weight for a dose of 50 mg/kg. Serial bleeding (0.2 ml) was performed via saphenous vein, alternating sampling from 3 animals for each time point. Blood was collected in lithium heparin plasma separator tubes and stored on ice until separated into plasma at 14,000 × *g* for 2 min. at 4 °C. Plasma samples were stored at -20 °C until analysis. Plasma samples were processed for analysis by thawing at room temperature followed by mixing with a tenfold v/v excess of methanol with subsequent filtration through 96-well Phenomenex Phree plates. The filtrates were analyzed by LCMS/MS on a Shimadzu LC-20AD HPLC system coupled to a Sciex API-5000 mass spectrometer. The analytical column (Agilent Poroshell C18; 2.1 × 100 mm) was eluted with a gradient of 5 to 100% Mobile Phase B over 11 min at 0.3 mL/min; Mobile Phase A: 95:5 H_2_O/CH_3_CN + 0.1% v/v formic acid; Mobile Phase B: CH_3_CN + 0.1% v/v formic acid. The LHF-535 concentration was determined by plotting the peak area of the mass spec transition of *m/z* = 413.2 to 353.2 for each plasma sample versus a standard curve from guinea pig plasma samples spiked with LHF-535 (500 to 50,000 ng/mL). All standards were diluted 1:100 after filtration through the Phree plate, whereas pharmacokinetic samples were diluted as necessary to fall within the concentration range of the standard curve. AUC_0–24 h_ (area under the time-concentration curve for the 24 h period after dosing) was calculated using the average plasma concentration for each time point and a combination linear-log trapezoidal method (linear for segments of the curve in which concentration increases and logarithmic for segments of the curve in which concentration decreases). AUC_0–24 h_ is the sum of the AUC from each of the segments comprising that time span.

### Statistical analysis

Appropriate group size was determined to be eight animals per group to ensure adequate (> 80%) statistical power. To determine statistically significant differences between treatment and control groups for plaque assay data, multiple, unpaired t-tests were performed on sequentially collected samples.

## Data Availability

Data analyzed in this work are available from the corresponding author on reasonable request.
